# Rescue of hyperexcitability in hippocampal CA1 neurons from Mecp2 ^(-/y)^ mouse through surface potential neutralization

**DOI:** 10.1371/journal.pone.0195094

**Published:** 2018-04-05

**Authors:** Saju Balakrishnan, Sergej L. Mironov

**Affiliations:** CNMPB (Centre for Nanoscale Microscopy and Molecular Physiology of the Brain, Cluster of Excellence 171, DFG Research Center 103), Institute of Neuro and Sensory Physiology, Georg-August-University, Göttingen, Germany; Universita degli Studi dell’Insubria, ITALY

## Abstract

Hyperventilation is a known feature of Rett syndrome (RTT). However, how hyperventilation is related to other RTT symptoms such as hyperexcitability is unknown. Intense breathing during hyperventilation induces hypocapnia and culminates in respiratory alkalosis. Alkalinization of extracellular milieu can trigger epilepsy in patients who already have neuronal hyperexcitability. By combining patch-clamp electrophysiology and quantitative glutamate imaging, we compared excitability of CA1 neurons of WT and Mecp2 ^(-/y)^ mice, and analyzed the biophysical properties of subthreshold membrane channels. The results show that Mecp2 ^(-/y)^ CA1 neurons are hyperexcitable in normal pH (7.4) and are increasingly vulnerable to alkaline extracellular pH (8.4), during which their excitability increased further. Under normal pH conditions, an abnormal negative shift in the voltage-dependencies of HCN (hyperpolarization-activated cyclic nucleotide-gated) and calcium channels in the CA1 neurons of Mecp2 ^(-/y)^ mice was observed. Alkaline pH also enhanced excitability in wild-type (WT) CA1 neurons through modulation of the voltage dependencies of HCN- and calcium channels. Additionally alkaline pH augmented spontaneous glutamate release and burst firing in WT CA1 neurons. Conversely, acidic pH (6.4) and 8 mM Mg^2+^ exerted the opposite effect, and diminished hyperexcitability in Mecp2 ^(-/y)^ CA1 neurons. We propose that the observed effects of pH and Mg^2+^ are mediated by changes in the neuronal membrane surface potential, which consecutively modulates the gating of HCN and calcium channels. The results provide insight to pivotal cellular mechanisms that can regulate neuronal excitability and help to devise treatment strategies for hyperexcitability induced symptoms of Rett syndrome.

## Introduction

The signaling pathways underlying the symptoms of Rett syndrome (RTT) are poorly understood. RTT is a neurodevelopmental disease caused by improper maturation of synapses as a consequence of the loss of expression of the Methyl-CpG binding protein-2, Mecp2 [1]. RTT phenotype (growth regression, neurological problems, motor deficits, and respiratory abnormalities) is emulated by a mouse model with a knock-out mutation of the corresponding gene (Mecp2^-/y^) [2]. Previously it has been shown that this mouse model demonstrates specific disturbances in calcium [3], cAMP [4, 5] and ATP homeostasis [6].

Isolated RTT mouse hippocampi are remarkably prone to epileptic seizures [6–9], which conform to the observations made in RTT patients. Clinical studies [10] show that RTT patients often suffer from breathing disturbances, such as irregular cycles of hyperventilation with concomitant hypocapnia. This is frequently followed by the development of apnoea (breath holding) and can lead to unconsciousness. Despite hyperventilation being a prominent feature of RTT, its impact on neuronal function is unclear. Hyperventilation can produce alkalosis, via hypocapnia and hypoxaemia which may have aberrant excitatory effects on brain and muscle activity [11, 12]. Such hyperexcitability is often an immediate cause of epileptic seizures and cramps.

The membrane potential of a cell divided into two components: the surface potential (SP), generated by putative negative charges of glycoproteins and extracellularly exposed anionic lipids on the cell membrane; and the transmembrane potential (TP). Effects of pH on neuronal activity can be mediated by changes in the surface potential that successively shifts the activation curves of voltage-sensitive ion channels [13, 14]. TP can change appropriately when SP is modified due to neutralization of negative charges by protons and divalent ions. Modulation of SP by shifts in external pH is translated into changes in TP, which accordingly modifies the voltage dependence of the sensors gating the ion channels. In addition, when surface potential becomes more negative, the current through the cation channels increase due to accumulation of permeating cations at the channel entrance [15], affecting the steady-state ion conductance. The excitability of cell membranes can thereby be perturbed by changes in the SP and through alteration of the voltage-dependent activation and permeability of ion channels. Neutralization of surface charges by cations can dampen the excitability and the unbinding of these screening ions may enhance excitability.

Alkalinization (decrease in external H^+^) promoting excitation and acidification (increase in H^+^) causing depression of activity can be attributed to SP modification. Divalent cations are more effective in neutralizing the charges, with additional ‘surface charge screening’ to stabilize the membrane excitability [14]. Changes in the SP of neuronal membranes can be assessed by measuring I-V relationship of the membrane channel conductance under varying pH or concentration of divalent cations in the extracellular environment.

Several research groups have reported elevated basal activity and augmented neuronal response to electrical stimulation, in RTT syndrome models [16–18]. We asked how enhanced vulnerability in RTT is expressed in respiratory alkalosis and which role the surface potential plays. Patch-clamp electrophysiology combined with calcium and glutamate imaging demonstrated that, alkaline pH enhanced glutamate release and augmented membrane channel activity in CA1 neurons in organotypic hippocampal brain slices. Exposure to acidic extracellular pH and elevated Mg^2+^ reduced glutamate release, attenuated membrane channel activity and suppressed excitability of RTT CA1 neurons. These changes occurred in parallel with the shifts in the activation curves of voltage-sensitive calcium (VCSS) and hyperpolarization-activated cyclic nucleotide-gated (HCN) channels. We focused on the role of these two classes of channels, as they operate in subthreshold membrane potentials and are shown to regulate the membrane excitability and spatial/temporal integration of inputs to neurons of various brain areas [19–21]. The activation curves of these channels moved either to hyperpolarizing (in alkaline pH) or to the depolarizing direction (in acidic pH and Mg^2+^) on the axis, which correlated appropriately with the presumed changes in surface potential. The findings suggest a simple mechanism that can regulate excitability in RTT, through the alteration of SP. The results can also interpret the previous empirical observation of magnesium treatment that greatly alleviated Rett symptoms [22].

## Materials and methods

### Animals

The animals were housed, cared for and euthanized according to the recommendations of the European Commission (No. L358, ISSN 0378–6978). Experimental protocols were approved by the Committee for Animal Research, Gottingen University. The mouse model for Rett syndrome; strain B6.129P2(C)—Mecp2 tm1-1Bird [2] obtained from the Jackson Laboratory (Bar Harbor, ME, USA) and maintained on a C57BL/6J background was used for experiments. Hemizygous mutant Mecp2^−/y^ males (RTT) were generated by crossing heterozygous Mecp2^+/−^ females with C57BL/6JWT males and genotyped to distinguish between the wild-type (WT) and RTT mice. Organotypic slices are prepared from littermate WT and Mecp2^−/y^ mice.

### Preparation of organotypic slices

The experiments in this study were done on organotypic hippocampal slices prepared as described previously using original protocol [23] modified in [6]. Briefly, the animals at postnatal day P3 were anesthetized with isoflurane and decapitated. The two hippocampi were isolated and 250-μm thick transverse slices were cut and placed on the support membranes (Millicell-CM Inserts, PICMORG50; Millipore). The surface of the slice was covered by a medium containing 50% MEM with Earle’s salts, and 25 mM HEPES, 6.5 mg/ml glucose, 25% horse serum, 25% Hanks solution buffered with 5 mM Tris and 4 mM NaHCO_3_, pH 7.3, changed every 2 days. It has been previously shown that RTT related abnormalities starts even earlier (at P3 to P9) than the appearance of overt symptoms at around P40 [3, 4, 24–29]. The age point of P3 was also selected because the survival percentage of neurons was declined when slices were prepared from older animals. The efficiency of the viral particles to transduce the slices was also comparatively high at early time points.

For the experiments, the slices were virally transduced with the glutamate sensor [30] targeted to neurons (AAV5.hSyn.iGluSnFr.WPRE.SV40) or astrocytes (AAV5.GFAP.iGluSnFr.WPRE.SV40). The constructs were purchased from Penn Vector Core, Department of Pathology and Laboratory Medicine; University of Pennsylvania, USA. Transduction was performed two days after plating slices, and sensor expression took usually one. The slices were used for experiments after 7 days in vitro (thus P10 to P24). Similar to our previous studies, where other genetically encoded sensors have been used [3, 6], no morphological and electrophysiological differences that distinguished neurons from their naive counterparts were noticed. Development of organotypic slices in vitro is shown to be similar to that of in-vivo cellular differentiation [3, 31]. The structural development and neuronal connections in organotypic slices were very similar to that of acute slices prepared from age matched animals.

In the experiments, the membrane with attached slice was fixed on a cover slip in the recording chamber and continuously perfused at 34 °C with artificial cerebrospinal fluid (ACSF) containing NaCl (138 mM), KCl (3 mM), CaCl_2_ (1.5 mM), MgCl_2_ (1 mM), HEPES (30 mM), NaH2PO4 (1 mM), glucose (10 mM), pH to 7.4 with NaOH. The volume of perfusion chamber was approximately 2 ml and the perfusion flow rate was 10 ml/min. Solutions were exchanged by the replacement of a distal reservoir by another one that contained drugs, which arrived at the chamber within 30 s. All salts and other common chemicals were from Sigma (Deisenhofen, Germany).

Approximately equal numbers of neurons were measured from WT and RTT mice and analyzed blind to genotype. Each test in this study was repeated with at least four different slice preparations. In total, 142 slices prepared from 26 WT and 158 slices from 23 RTT mice were analyzed in this study. The networks of astrocytes and neurons with different morphological appearances were depicted by appropriate sensor expression. It was yet impossible to precisely localize the source of extracellular glutamate reported by these sensors. The plasma membranes of apposing neurons or astrocytes are normally separated by less than 0.1 μm, which is beyond the diffraction limit. Variations could not be detected even by imaging with acquisition times as fast as 2 ms. Representative traces are shown in S1 Fig. During analysis we pooled the data obtained from neuronal and glial glutamate sensors. The mean data in imaging experiments were routinely obtained from 12 neurons present in the image field per slice. The number of neurons examined in patch-clamp experiments and the statistical significance of the data are indicated in respective ‘Results sections or Figure legends’.

### Imaging

The neurons were viewed under a 40× water immersion objective (LUMplanFI, N. A. 0.8). The optical recording system consisted of an upright microscope (BX51, Olympus, Hamburg, Germany) equipped with a monochromator (Optoscan, CAIRN, Kent, UK). Images from a cooled CCD camera (ANDOR) were digitized (256×256 pixels at 12 bit resolution) and acquired with ANDOR software. Excitation/emission wavelengths for glutamate imaging with iGluSnFR were 470/525±10 nm (49002, Chroma technology, Olching, Germany). In a set of experiments, the cells were filled with 100 μM fluo-4 (Thermo Fisher, Germany) to image intracellular calcium, and the same filter combination as above was used. To visualize neuron morphology, 100 μM Alexa 568 (Thermo Fisher, Darmstadt, Germany) was added to the patch pipette. Its fluorescence was excited at 565 nm and the emission was separated from the excitation light with dichroic mirror centered at 585 nm (T585lpxr, Chroma Technology, Olching, Germany), and collected through 610/75 nm filter (45186, Chroma Technology, Olching, Germany). Calibrations showed that iGluSnFr reports ambient glutamate levels from 1 μM to 1 mM. The relative changes in fluorescence were quantified by modified Michaelis-Menten equation, Δ*F/Fo* = *Fmax* [*Glu*]/(*Kd* + [*Glu*]) where *F*_*o*_ is the resting (background-subtracted) fluorescence level, and the values of *F*_*max*_ and *K*_*d* =_ 10 μM were obtained from calibrations.

### Electrophysiology

Patch electrodes of borosilicate glass (WPI, Berlin, Germany) had resistances of 2–3 MΩ when filled with intrapipette solution containing: K^+^-gluconate (110 mM), KCl (5 mM), HEPES (50 mM), EGTA (0.005 mM), MgSO_4_ (4 mM), ATP (4 mM), GTP (0.2 mM), phosphocreatine (9 mM); pH 7.4 (titrated with 1 M KOH). Alternatively a Cs^+^ based solution contained CsMeSO_4_ (92 mM), CsCl (43 mM) TEA-Cl (5 mM), EGTA (0.4 mM), MgCl_2_ (1 mM), HEPES (10 mM), ATP (4 mM), GTP (0.4 mM); pH 7.4 (titrated with CsOH) was used for HCN and calcium current recordings. Patch-clamp recordings were done with an EPC-9 amplifier (HEKA, Ludwigshafen, Germany) as described previously [5]. Membrane currents were filtered at 3 kHz (−3 dB), digitized at 10 kHz, and stored for off-line analysis. Series resistance ranged from 10–15 MΩ and was constantly monitored. Cells showing more than ten percent fluctuation in series resistance were discarded from the analysis. HCN and VSCC currents were evoked by voltage steps to -110 and -40 mV, respectively, from a holding potential of -70 mV (close to resting potential measured in CA1 neurons). No significant differences in the amplitude or kinetics of the evoked currents were observed after blockade of neuronal or synaptic activity. The activation curves were obtained from the amplitude of HCN and VSCC tail currents. Pure capacitance transients were partially removed by on-line compensation and further subtraction from the tail currents was done numerically off-line. The passive transients were approximated by a smooth exponential pattern and subtracted from the records after appropriate scaling.

### Data analysis

Data were analyzed using Patchmaster software (HEKA Electronics). Imaging data were analyzed using Metamorph software (Princeton Instruments). Statistical significance was determined by using the paired Student’s t test (within-group comparison of paired events), and the Mann–Whitney U test (between-group comparison), when appropriate, with *P* < 0.05 being the criterion for statistical significance. All data are shown as mean ± SEM. Power of the sample sizes (minimum 80%) were calculated using Origin 8 software (Massachusetts, USA). Action potential (AP) kinetics was also analyzed using Origin 8. APs from WT and RTT neurons were analyzed to compare threshold, rise and decay times. APs before and after the change of pH or application of 8mM Mg^2+^ were analyzed to monitor the effects induced by these applications.

## Results

### Extracellular alkalinization enhances excitability of CA1 neurons

To imitate the effects of respiratory acidosis and alkalosis, the hippocampal slices from WT and RTT mice were perfused with acidic or alkaline ACSF, while measuring the CA1 neuron activity, using whole cell patch-clamp. One unit shifts in the pH (6.4 and 8.4) from a normal pH of 7.4 were selected to impose clearly observable effects. These values are similar to the pH variations measured in the brain [12].

Basal electrophysiological properties of WT and RTT neurons were routinely examined at the beginning of patch clamp experiments. The resting membrane potentials of CA1 neurons from WT (-71.8 ± 3.34 mV, n = 60) and RTT (-72.37 ± 3.78 mV, n = 60) slices showed no statistically significant differences (*P* = 0.69, Mann-Whitney U test, S2A Fig). Similarly, whole cell capacitance of WT (117.50 ± 3.2 pF, n = 48) and RTT CA1 (114.62 ± 2.4 pF, n = 54) neurons also did not show statistically significant differences (*P* = 0.97, Mann-Whitney U test, S2B Fig).

**Fig 1:**
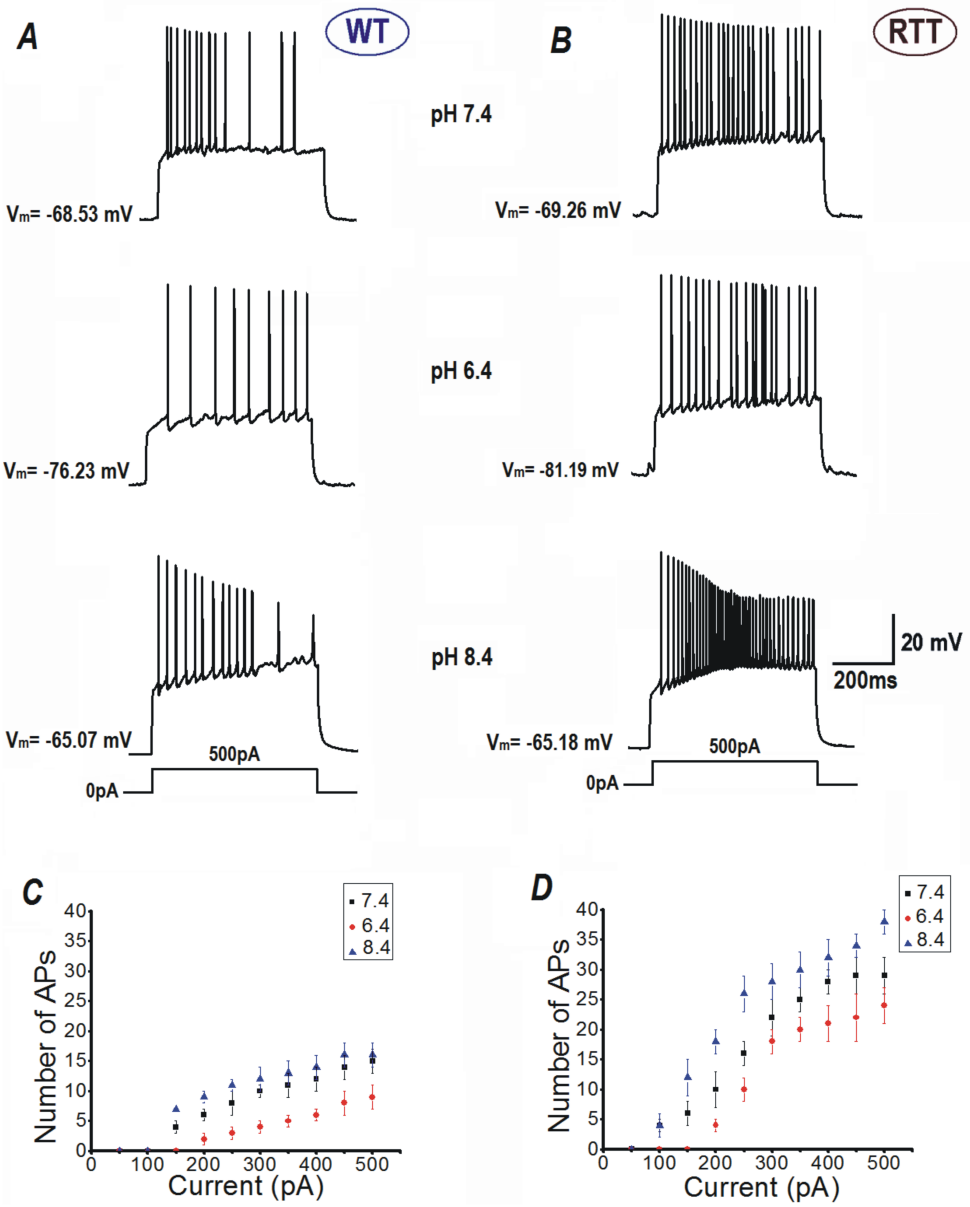
Outer surface potential modulates excitability in CA1 neurons after changes in extracellular pH. Representative responses of CA1 neurons from hippocampus of WT (**A**) and RTT animals (**B**) to current injections. Note the clear differences between responses recorded in the acidic (suppression of activity) and alkaline ACSF (activation) from that in the control. **C** and **D** Input-output relationships of CA1 neurons from each genotype. The number of APs fired vs. intensities of current injection.

The input-output relationships of CA1 neurons in response to current injection were differentially modulated by extracellular pH (Fig 1). CA1 neurons from WT slices fired (upon 500 ms long pulses) on average 14.5 ± 0.76 APs and showed evident spike rate adaptation (Fig 1A top, and Fig 1C, n = 55), in response to a current injection of 500 pA to evoke membrane depolarization. Exposure to acidic solution (pH 6.4) decreased the number of action potentials to 7.6 ± 0.65 (Fig 1A middle and Fig 1C, n = 16, *P*<0.05, Student’s t test); whereas, perfusion with alkaline ACSF (pH 8.4) increased their number only to 15.9 ± 0.9 (Fig 1A bottom and Fig 1C, n = 19, *P =* 0.14, Student’s t test) with the same current injection.

In comparison to WT CA1 neurons, naïve RTT CA1 neurons demonstrated higher intrinsic excitability and generated much more APs on current injections. On injection with a current of 500 pA, RTT CA1 neurons fired on average 27.6 ± 1.5 APs (Fig 1B top, Fig 1D, n = 59, *P*<0.01, Mann-Whitney U test) at pH 7.4, with reduced spike rate adaptation, unlike WT CA1 neurons. ACSF with pH 6.4 suppressed the excitability of RTT neurons significantly and the number of APs decreased to 21.6 ± 1.7. (Fig 1C middle, Fig 1D, n = 24, *P*<0.05, Student’s t test). This was still significantly higher than the output of WT CA1 neurons at pH 6.4 (*P*<0.001, Mann-Whitney U test) and pH 7.4 (*P*<0.05, Mann-Whitney U test). Contrary to that in WT CA1 neurons, the AP output of RTT CA1 cells was significantly augmented further by perfusion of ACSF with alkaline pH (8.4) to 37.8 ± 2.4 (Fig 1C bottom, Fig 1D, n = 24, *P*<0.05, Student’s t test), indicating further excitability and lack of adaptation. The rate of RTT CA1 neuron AP output at pH 8.4 was also statistically higher to that of WT neurons at pH 8.4 (*P*<0.01, Mann-Whitney U test).

The input resistance of RTT CA1 (149.82 ± 7.37 MΩ, n = 48) neurons did not statistically differ (*P* = 0.09, Mann-Whitney U test) from that of WT CA1 neurons (168.46 ±11.15 MΩ, n = 54) as shown in S1C Fig. Application of ACSF with pH 6.4 reduced the input resistance of RTT neurons (n = 24) significantly (*P*<0.05, Student’s t test) to 104.81 ± 3.18 MΩ, S1C Fig). Perfusion with ASCF at pH 8.4 did not change the input resistance of RTT neurons significantly (134.72 ± 13.84 MΩ, *P* = 0.12, n = 24, Student’s t test, S1C Fig).

Rise times of single APs (S3A and S3E Fig) of WT (0.8 ± 0.011 ms, n = 25) and RTT (0.7 ± 0.01 ms, n = 25) CA1 neurons did not differ substantially (*P =* 0.62, Mann-Whitney U test). However, RTT CA1 neurons (2.7 ± 0.04 ms) had distinctively faster AP decay times (S3A and S3F Fig) than WT CA1 neurons (3.9 ± 0.03 ms, *P*<0.05, Student’s t test). Exposure to ACSF with pH 6.4 (S3B and S3E Fig, n = 24) significantly increased the AP rise time of RTT CA1 neurons (1.2 ± 0.02 ms, *P*<0.05, Student’s t test) in comparison to control AP rise times. The decay time (S3B and S3F Fig) was also significantly increased at pH 6.4 (3.4 ± 0.0 ms, *P*<0.05, Student’s t test). Application of ACSF with pH 8.4 (S3C and S3E Fig, n = 24) did not change the AP rise time (0.6 ± 0.12 ms, *P* = 0.12, Student’s t test) or decay time (2.3 ± 0.03 ms, *P* = 0.08, Student’s t test, S3C and S3F Fig) of RTT CA1 neurons.

AP threshold was marginally lower in RTT CA1 neurons. WT CA1 neurons had a mean AP threshold of -42.9 ± 0.26 mV (n = 25) and RTT CA1 neurons showed an AP threshold of -44.67 ±0.92 mV, (n = 25, *P* = 0.63, Mann-Whitney U test). Exposure to ACSF with pH 6.4 (S3B and S3G Fig, n = 24) shifted the AP threshold of RTT CA1 neurons significantly to more positive value (-28.84 ± 0.3 mV *P*<0.05, Student’s t test). The increase in pH to 8.4 (S3C and S3G Fig, n = 24) did not effectively change the threshold of APs in the RTT CA1 neurons (-41.95 ± 0.36 mV, *P =* 0.11, Student’s t test).

Even though the basal electrophysiological properties did not drastically differ between WT and RTT CA1 neurons, the latter showed higher intrinsic excitability. It was also observed that the RTT CA1 neurons were more susceptible to alkaline environment induced effects.

### Voltage-dependent activation of I_HCN_ and I_VSCC_ is shifted to negative potential in RTT

**Fig 2:**
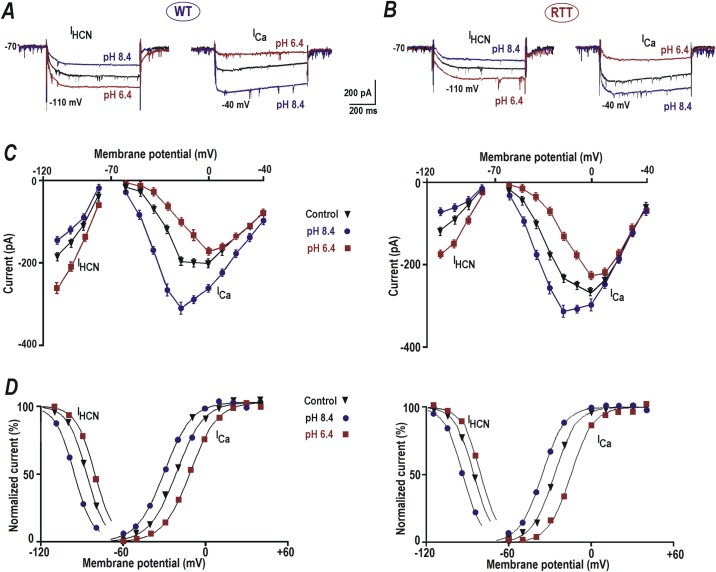
Modulation of HCN and calcium currents by extracellular pH. ***A*** and ***B***–Sample HCN and VSCC currents from WT and RTT respectively. The currents were evoked by voltage steps to -110 and -40 mV, respectively, from a holding potential of -70 mV (close to resting potential measured in CA1 neurons). The cells were patched using Cs^+^ + TEA intracellular solution (see Methods) that allowed clear isolation of the hyperpolarization-activated (I_HCN_) and depolarization-evoked calcium (I_Ca_) currents; which were activated below -70 and above -60 mV, respectively. HCN current was smaller and VSCC current was bigger in RTT CA1 neurons in comparison to WT (***A*** and ***B***, black traces). A decrease in pH to 6.4 increased the HCN current (***A*** and ***B*** left, brown traces) and reduced the Ca^2+^ current (***A*** and ***B*** right, brown traces). pH increase to 8.4 caused a reduction in HCN current (***A*** and ***B*** left, blue traces) and potentiation (***A*** and ***B*** right, blue traces) of the calcium current. ***C*** and ***D—***I-V curves for steady state HCN and VSCC currents from WT and RTT CA1 cells. ***E*** and ***F—***Activation curves (*m*_*∞*_) determined from the tail currents. The points represent the means measured in four CA1 cells from WT and RTT slices. The data were mean-square fitted with the Boltzmann function *m*_*∞*_ *= 1*/ [*1 + exp* (-(*V—V*_*1/2*_)/*b*)]. The slope factor of *b =* 8 mV was found to be the same for HCN and VSCC currents.

The data in Fig 1 suggest the role of surface potential in determining excitability patterns of WT and RTT neurons. Therefore, we examined for changes in the gating of two classes of subthreshold conductances viz. hyperpolarization-activated cyclic nucleotide-gated (HCN) channels and voltage-sensitive calcium channels (VSCC). At physiological pH (7.4), WT neurons (n = 32) showed an average I_HCN_ amplitude of -198.74 ± 8.2 pA on hyperpolarization to -110 mV (Fig 2A and 2C, left, black traces) from a holding potential of -70mV. pH decrease to 6.4 significantly enhanced the amplitude of I_HCN_ to -267.38 ± 12.7 pA for the same hyperpolarization step (Fig 2A and 2C, left, brown traces, n = 15, *P*<0.05, Student’s t test). Conversely, an increase in pH to 8.4 diminished the I_HCN_ amplitude significantly to -151.41 ± 10.36 pA upon hyperpolarization to -110 mV (Fig 2A and 2C, left, blue traces, n = 19, *P*<0.05, Student’s t test).

WT CA1 neurons displayed basal I_Ca_ amplitude of -65.17 ± 6.4 pA on depolarization to -40 mV from the holding potential of -70 mV (Fig 2A and 2C, right, black traces, n = 30). Application of ACSF with pH 6.4 significantly depressed the amplitude of I_Ca_ to -22.17 ± 4.2 pA in response to the depolarization step to -40 mV (Fig 2A and 2C, right, brown trace, n = 20, *P*<0.05, Student’s t test). Perfusion of slices with ACSF of pH 8.4, on the contrary, enhanced the amplitude of I_Ca_ significantly to -112.28 ± 5.8 pA (Fig 2A and 2C, right, blue traces, n = 22, *P*<0.05, Student’s t test).

In CA1 neurons of RTT slices, hyperpolarizing step to -110 mV evoked I_HCN_ with an average amplitude of -116.64 ± 7.2 pA (Fig 2B and 2D, left, black traces, n = 32), which was significantly lower than in the WT CA1 neurons (*P*<0.05, Mann-Whitney U test). At extracellular pH 6.4 I_HCN_ amplitude mean enhanced significantly to -174.64 ± 4.3 pA (Fig 2B and 2D, left, brown traces, n = 19, *P*<0.05, Student’s t test) but was still significantly lower than the amplitude of I_HCN_ observed in WT CA1 neurons at acidic pH (*P*<0.05, Mann-Whitney U test). Elevation of extracellular pH to 8.4 had the opposite effect and significantly reduced the mean amplitude of I_HCN_ to -73.33 ± 4.1 pA (Fig 2B and 2D, left, blue traces, n = 21, *P*<0.05, Student’s t test), which was significantly lower than the amplitude observed in WT CA1 neurons at pH 8.4 (*P*<0.05, Mann-Whitney U test).

In RTT CA1 neurons, the depolarizing step to -40 mV from a holding potential of -70 mV induced an evidently higher I_Ca_ current with mean amplitude of -98.79 ± 3.9 pA (Fig 2B and 2D, right, black traces, n = 35, *P*<0.05, Mann-Whitney U test) to that of WT CA1 neurons. Exposure to acidic pH (6.4) suppressed the amplitude to -49.46 ± 3.6 pA (Fig 2B and 2D, right, brown traces, n = 24). This was nonetheless significantly bigger to that measured in the WT CA1 neurons at acidic pH (*P*<0.05, Mann-Whitney U test). In contrast, application of alkaline pH (8.4) still elevated the I_Ca_ amplitude to -173.51 ± 4.2 pA (Fig 2B and 2D, right, blue traces, n = 25); again showing significantly higher augmentation than in the WT neurons (*P*<0.05, Mann-Whitney U test).

The data thus indicate that, in comparison to WT CA1 neurons, RTT CA1 neurons displayed significantly reduced basal I_HCN_ (1.70 ± 0.07 times smaller, *P*<0.05, Mann-Whitney U test) currents and a significantly larger I_VSCC_ current (1.51 ± 0.06 times bigger, *P*<0.05, Mann-Whitney U test) These variations in conductance may have contributed to the increased excitability displayed by RTT CA1 neurons.

The variations in the I-V curves (Fig 2D) of I_HCN_ and I_Ca_ in CA1 neurons of RTT and WT slices can be explained by the shifts of corresponding VSCC and HCN activation curves (Fig 2E and 2F). They were obtained from the corresponding tail currents. For both HCN and VSCC channels in CA1 neurons (n = 30) from naïve RTT slices, the mid-point of activation (*V*_*1/2*_) was significantly more negative (6.17 ± 0.4 mV, n = 12, *P*<0.05, Mann-Whitney U test) than in WT (Fig 2F, black traces). Perfusion of ACSF with pH 6.4 shifted the activation curves of HCN and VSCC currents into the depolarizing direction, and ACSF with pH 8.4 induced the opposite effect (Fig 2D), both in WT and RTT CA1 neurons. The mid-points for the activation of HCN currents at extracellular pH 7.4 (control), 6.4 and 8.4 were *V*_*1/2*_ *=* -81.5 ± 0.6, -76.3 ± 0.5 and -89.4 ± 0.7 mV (WT, n = 14) and *V*_*1/2*_ *=* -87.4 ± 0.6, -81.7 ± 0.5 and -96.1 ± 0.7 mV (RTT, n = 17), respectively and were significantly more negative shifted in RTT CA1 neurons (*P*<0.05, Mann-Whitney U test). For VSCC currents the mid-points were *V*_*1/2*_ *=* -21.3 ± 0.5,-11.7 ± 0.4 and -30.1 ± 0.6 mV (WT, n = 14) and *V*_*1/2*_ *=* -27.2 ± 0.6, -15.4 ± 0.5 and -35.6 ± 0.4 mV (RTT, n = 17), respectively, displaying that the activation curves of I_ca_ were also significantly negative-shifted in RTT CA1 neurons (*P*<0.05, Mann-Whitney U test).

The data indicate that the magnitude and activation properties of HCN and VSCC channels favor elevated excitability in the RTT CA1 neurons. Alkaline extracellular condition aggravated this further by shifting the activation curves towards the resting membrane potentials.

### Alkalinization enhances glutamate release

**Fig 3:**
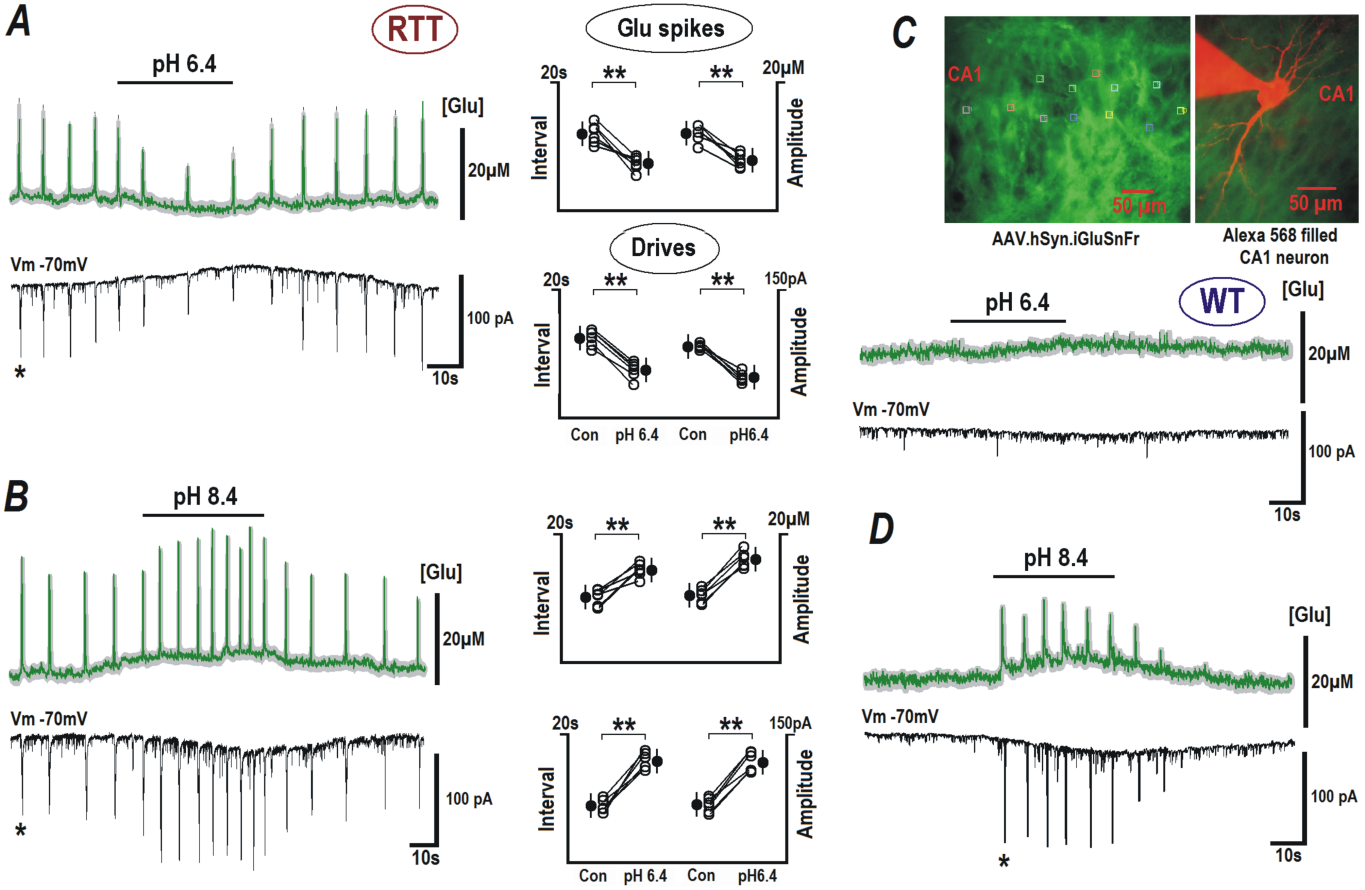
Modulation of glutamate spikes and bursting activity by extracellular pH. RTT and WT slices were transduced with neuron-targeted virus with glutamate sensor iGluSnFr (see Materials and methods). Upper traces in each panel indicate mean glutamate changes (obtained from 12 neurons in the image field) overlaid upon grey background indicating ± SEM. Brief glutamate transients in RTT slices appeared synchronously in the image field. They temporally coincided with the synaptic drives (a correlate of the bursting activity) in the patched CA1 cell (lower block traces in each panel). ACSF with pH 6.4 suppressed the generation of glutamate spikes and the synaptic currents (***A***). Elevation of pH to 8.4 increased the frequency and amplitude of glutamate spikes and corresponding synaptic currents (***B***). Group summary for experiments in ***A*** and ***B*** is presented on the right. The data for RTT were evaluated before and after pH effects stabilized (ca. 2 min), with Student’s t test with confidence levels of *P* < 0.01 (**). In the case of WT, neither glutamate levels nor synaptic activity were modified at acidic pH (6.4, ***C***), but during the application of alkaline ACSF with pH 8.4, glutamate spikes and synaptic drives appeared (***D***). The inset in the right upper corner shows the sensor fluorescence in CA1 area and the image of patched neuron filled with 100 μM Alexa 568 (red) overlaid on the image of neuronal glutamate sensor in the slice (green).

To study how external pH changes affect glutamate handling, patch-clamp recordings were combined with the imaging of ambient glutamate in the CA1 area, with the glutamate sensor iGluSnFr. Surprisingly we regularly observed spontaneous glutamate transients in the slices from RTT animals. Their average amplitude was 18.02 ± 1.5 μM and they occurred at a mean interval of 8.74 ± 1.4s (n = 60). These events were seldom in the WT (see Supporting Information for details). These glutamate spikes temporally matched the rhythmic bursting activity in the form of repetitive synaptic drives (recorded in the whole-cell mode) in the CA1 neurons of RTT mice (Fig 3A, 3B and 3D). Perfusion of ACSF with acidic pH (6.4) significantly suppressed the amplitude of glutamate spikes (green trace) to 7.91 ± 1.3 μM (*P*<0.05, Student’s t test) and increased the interval between these spikes to 12.22 ± 1.3 s (*P*<0.05, Student’s t test). The amplitude of synaptic drives (black trace) also reduced in response to acidic pH from -100.95 ± 2.65 to -56.03 ± 2.35 pA (*P*<0.05, Student’s t test) and the interval between the synaptic drives increased from 8.02 ± 0.7 to 12.05 ± 1.1s (*P*<0.05, Student’s t test) in RTT CA1 neurons (Fig 3A, n = 14).

Alkaline ACSF (pH 8.4) had an additional stimulatory effect on the glutamate spiking and bursting of RTT slices (Fig 3B, n = 14). As a result, the amplitude of glutamate spikes in RTT CA1 neurons increased significantly from 10.14 ± 0.9 to 18.27 ± 0.8 μM (*P*<0.05, Student’s t test) and the interval between the spikes reduced significantly from 14.83 ± 1.0 to 10.05 ± 0.9 s (*P*<0.05, Student’s t test). Similarly the amplitude of the corresponding EPSCs increased from -68.16 ± 2.64 to -117.43 ± 2.46 pA (*P*<0.05, Student’s t test) and the interval between them decreased from 18.14 ± 1.2 to 9.37 ± 1.1 s (*P*<0.05, Student’s t test). In WT cells, the exposure to acidic ACSF was without any effect (Fig 3C, n = 10), whereas ACSF with pH 8.4 had stimulated the generation of glutamate spikes. They reached the mean amplitude of 12.6 ± 0.9 μM and an interspike interval of 12.2 ± 0.7 s; and synaptic drives appeared with mean peak amplitudes of -77.22 ± 3.42 pA and interval of 11.8 ± 08 s, (Fig 3D, n = 10).

Thus, CA1 in RTT slices demonstrated substantially different glutamate handling that reflected higher spontaneous neuronal activity. In the WT slices, glutamate spikes were rarely seen (much lower amplitude and frequency when observed). Notably, alkaline pH converted the glutamate handling in WT slices to that observed in RTT slices. Moreover, the increase in pH was still able to exacerbate the spontaneous activity in RTT slices.

### Magnesium increase dampens hyperexcitability in RTT

Protons and divalent cations modulate the properties of ion channels through alteration of surface potential by screening/binding to the membrane lipids and glycoproteins [13, 15, 32]. Increasing Ca^2+^ can have deleterious effects on RTT CA1 neurons, by enhancing glutamate release [33, 34], and it is unwise to use Ca^2+^ to neutralize the surface charges. Mg^2+^ on the contrary is demonstrated to be equally effective [15]. Importantly, it is also shown to alleviate the symptoms of RTT patients [22] with minimal side effects. Therefore we tested whether Mg^2+^ increase can reduce the uncontrollable firing in RTT CA1 neurons. It is previously shown that an extracellular concentration of 4–8 mM Mg^2+^ alters neuronal activity [35].

**Fig 4:**
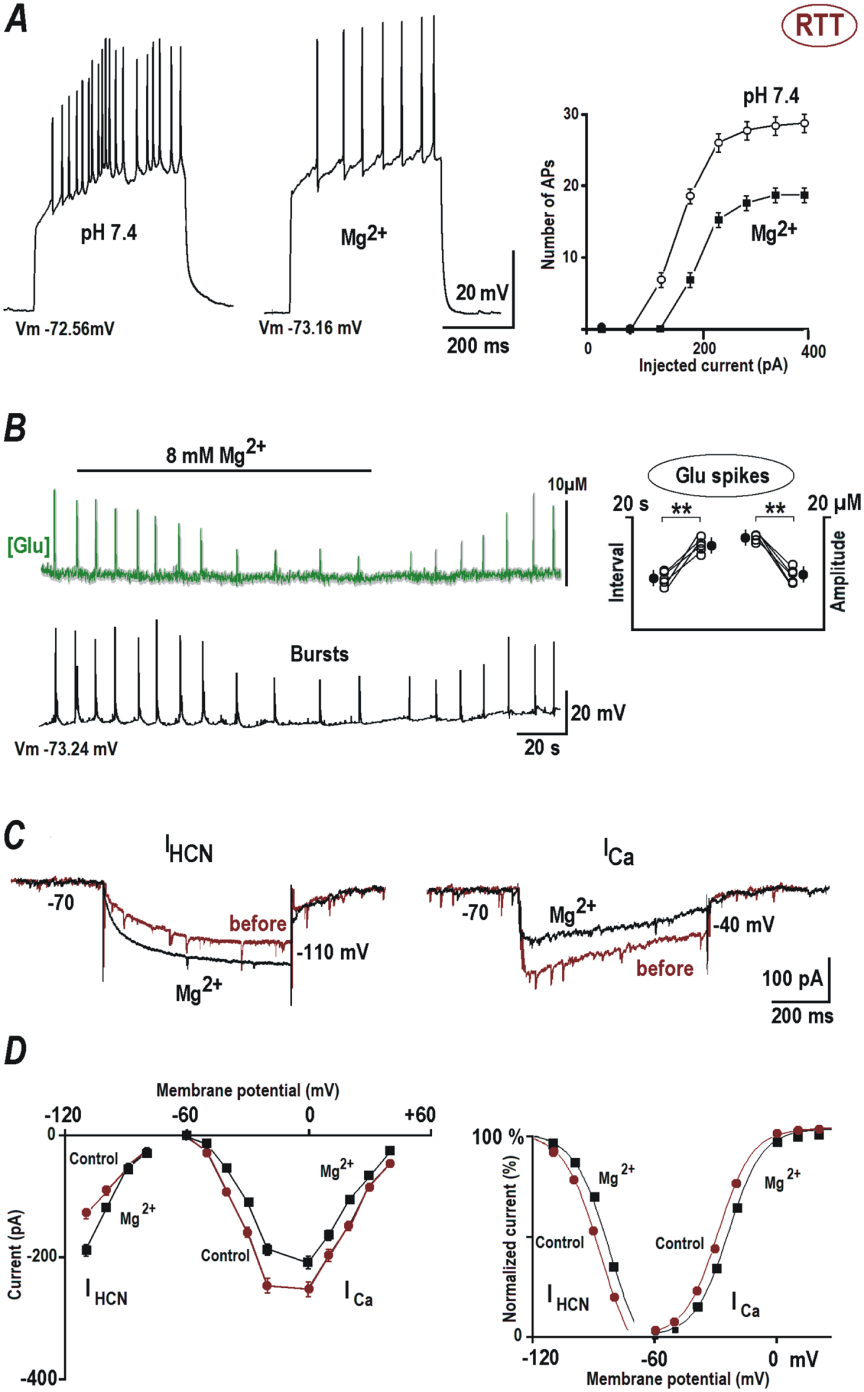
The action of magnesium on HCN and calcium channels, glutamate spikes and EPSCs are mediated by changes in the surface potential. ***(A***) Responses of CA1 neurons from RTT animals to current injections. 8 mM Mg^2+^ reduced the number of evoked APs. The input-output relationships on the right present mean data obtained from eight CA1 cells examined in 4 different preparations. (***B*)** Mg^2+^ diminished the amplitude and frequency of glutamate spikes (upper trace) and AP bursts (lower black trace). Glutamate changes are shown as an average (obtained from 12 neurons in the image field) overlaid upon grey background indicating ± SEM. The data were evaluated (Student’s t test with confidence levels *p* < 0.01 (**)) before and after Mg^2+^ effect stabilized (ca. 2 min) and presented in the group summary on the right. (***C)*** Mg^2+^ effects on HCN- and calcium currents. The currents were evoked by voltage steps to -110 and -40 mV, respectively, from the holding potential -70 mV. (***D*)** I-V curves were obtained for steady state currents and the activation curves (***D***) were determined from the tail currents. The data was approximated by the Boltzmann function *m*∞ = 1/ [1 + *exp* (−(*V* − *V*1/2)/*b*)] with the same slope factors for HCN-currents and the calcium currents (8 mV).

Elevation of Mg^2+^ concentration to 8 mM in the ACSF had a stabilizing effect on the excitability of CA1 neurons from RTT. The input resistance (145.28 ± 23.58 MΩ) was not altered significantly (S1C Fig, *P* = 0.09, Student’s t test), as well as the input-output relationship (in Fig 4A) was akin observed at pH 6.4 (Fig 1). Stimulation with 200 pA current injection (left traces in Fig 4A) induced on average 19.5 ± 2.4 APs in the RTT CA1 neurons at normal pH conditions. Perfusion with ACSF containing 8 mM Mg^2+^ significantly reduced the number of APs to 7.64 ± 2.3 in response to the same current injection (n = 16, *P*<0.05, Student’s t test). Application of 8 mM Mg^2+^ (S3D and S3E Fig, n = 16) resulted in a significant increase in the AP rise time of RTT CA1 (0.1 ± 0.01 ms, *P*<0.05, Student’s t test) and in the decay time (3.7 ± 0.02 ms, *P*<0.05, Student’s t test, S3D and S3F Fig). 8 mM Mg^2+^ application (S3D and S3G Fig, n = 16) also resulted in a significant shift in the AP threshold of RTT CA1 neurons to a more positive value (-29.72 ± 0.45 mV, *P*<0.05, Student’s t test).

In glutamate imaging experiments, Mg^2+^ suppressed the glutamate spike amplitude (from 17.62 ± 0.6 to 9.63 ± 0.5 μM, *P*<0.05, Student’s t test), increased the interspike intervals (from 16.82 ± 0.7 to 9.81 ± 0.5 s) and decreased bursting activity of RTT CA1 neurons (Fig 4B, n = 5, *P*<0.05, Student’s t test).

The whole cell recordings showed an increase in the amplitude of HCN current in response to hyperpolarizing voltage steps (from -109.74 ± to -197.84 ± 5.8 pA, n = 14, *P*<0.05, Student’s t test) and a decrease in the amplitude of calcium current (from -96.14 ± 2.8 to -44.57 ± 2.2 pA, n = 14, *P*<0.05, Student’s t test) in presence of augmented Mg^2+^ concentration (Fig 4C and 4D). We observed a rightward (depolarizing) shift in the activation curves of HCN and VSCC currents (Fig 4D, right), in accordance with the neutralization of the surface potential by Mg^2+^. The mid-points for HCN-current activation before and after Mg^2+^ addition were *V*_*1/2*_ *=* -87.1± 0.6 and -82.7 ± 0.5 mV (n = 14, *P*<0.05, Student’s t test) and for the calcium currents *V*_*1/2*_ *=* -29.6 ± 0.6 and -24.7 ± 0.6 m (n = 15, *P*<0.05, Student’s t test), respectively.

The data indicate that increasing magnesium had an alleviating effect on the hyperexcitability of RTT CA1 neurons without altering the input resistance. We presume that magnesium neutralizes the surface charges of these neurons and thus shifts the activation curves of subthreshold channels responsible for determining neuron’s excitability.

### Effects of surface potential on synaptic activity

Both pre- and postsynaptic HCN and VSCC channels are actively involved in glutamate release [36, 37] and in the processing of synaptic inputs [38–40]. To unravel roles of these channels more clearly, we analyzed the mean synaptic currents recorded in control conditions, during pH changes and at elevated Mg^2+^ in RTT and WT neurons.

**Fig 5:**
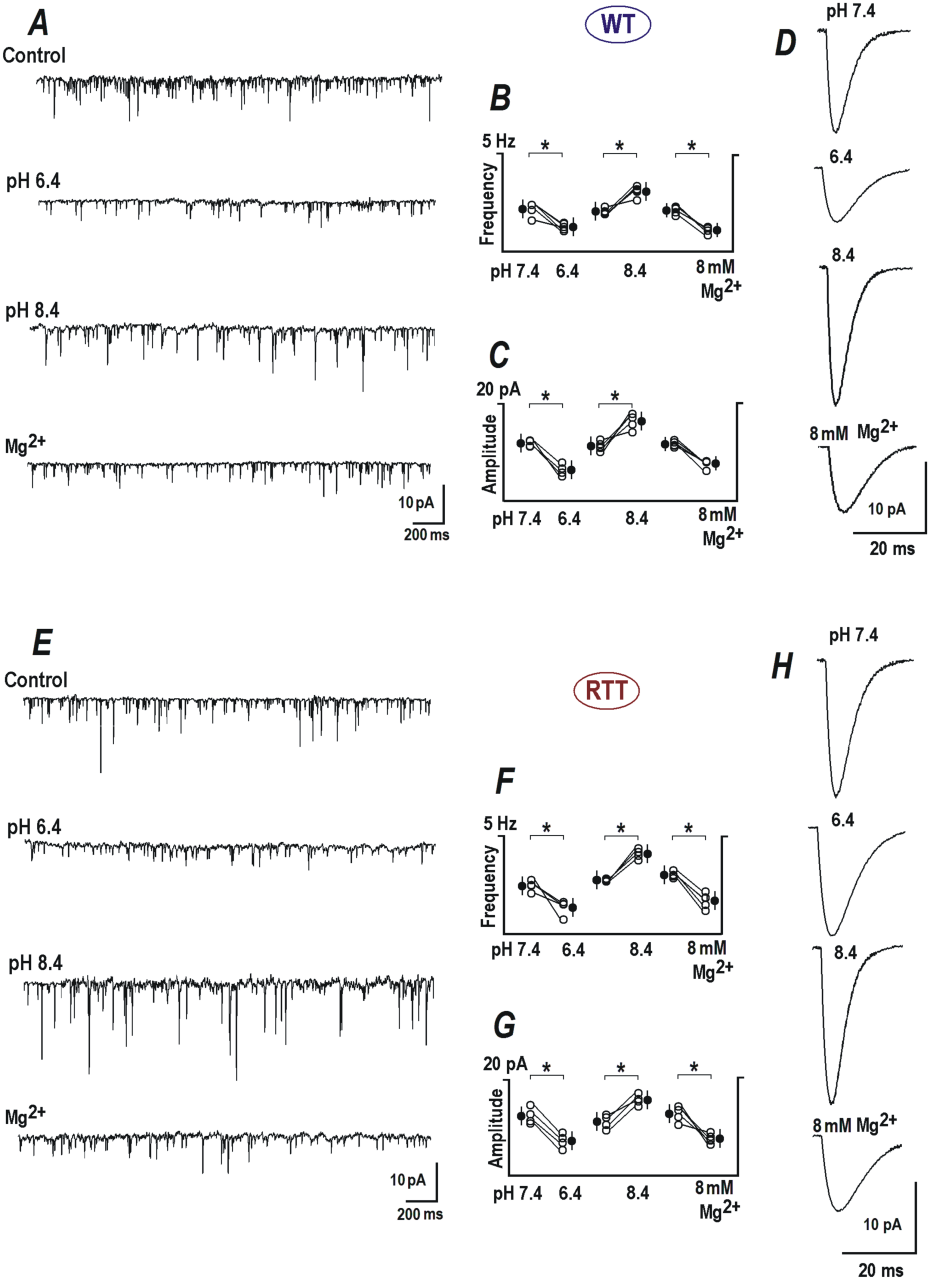
Modulation of EPSCs by extracellular pH. (***A***) Miniature excitatory synaptic currents (mEPSCs) measured in CA1 neurons from WT. Samples were measured before and 2 min after application of ACSF with pH 6.4 and 8.4, and 8 mM Mg^2+^, respectively. Group summaries (middle) are indicative of both pre- and postsynaptic effects because we observed changes both in frequency (***B***) and amplitude of minis (***C***). (***D***) Mean EPSCs obtained from averaging 20 to 30 single mEPSCs recorded under the specified conditions. (***E***) Representative mEPSCs traces from RTT CA1 neurons under control, pH 6.4, 8.4 and in presence of 8 mM Mg^2+^. Group analysis of frequency (***F***) and amplitude (***G***) during individual experimental conditions are shown in the middle. (***H***) Mean representative mEPSCs from RTT CA1 neurons during the indicated conditions are shown in the right panel.

mEPSCs were recorded in presence of TTX (0.5 μM) from WT and RTT CA1 neurons (Fig 5, 12 neurons each). In WT CA1 neurons, miniature release events appeared with a mean frequency of 2.39 ± 0.63 Hz and average amplitude of 8.03 ± 2.1 pA (Fig 5A, 5B, 5C and 5D) during control measurements at an external pH of 7.4. In ASCF with pH 6.4, mEPSCs had much lower frequency (1.35 ± 3.2 Hz, *P*<0.05, Student’s t test, Fig 5A and 5B) and smaller amplitude (4.87 ± 2.3, *P*<0.05, Student’s t test, Fig 5A, 5C and 5D) and the opposite effects were produced by alkalinization, where the frequency was elevated to 2.65 ± 0.7 (*P*<0.05, Student’s t test, Fig 5A and 5B) and the amplitude increased to 14.27 ± 2.2 pA (*P*<0.05, Student’s t test, Fig 5A, 5C and 5D).

In RTT CA1 cells, mEPSCs occurred at a basal rate of 2.54 ± 0.62 Hz (Fig 5E and 5F), and an amplitude of 14.35 ± 2.8 pA (Fig 5E and 5G) in. Exposure to acidic ACSF reduced the frequency of the RTT mEPSCs to 1.41 ± 0.73 Hz (*P*<0.05, Student’s t test, Fig 5E and 5F) and the amplitude to 6.42 ± 2.1 pA (*P*<0.05, Student’s t test, Fig 5E, 5G and 5H). ACSF with pH 8.4 increased the frequency of mEPSCs to 3.97 ± 0.84 Hz (*P*<0.05, Student’s t test, Fig 5E and 5F) and the amplitude to 17.78 ± 2.3 pA (*P*<0.05, Student’s t test, Fig 5E, 5G and 5H).

8 mM Mg^2+^ had similar effects to that of acidification, on the kinetics of mEPSCs of WT and RTT CA1 neurons. In the WT, 8 mM Mg^2+^ reduced the frequency from 2.21± 0.68 to 1.22 ± 0.52 Hz (*P*<0.05, Student’s t test, Fig 5A (Mg^2+^) and Fig 5B) and the amplitude from 7.57 ± 2.1 to 4.34 ± 1.6 pA (*P<*0.1, Student’s t test, Fig 5A (Mg^2+^), Fig 5C and 5D). Correspondingly, 8 mM Mg^2+^ decreased the frequency of mEPSCs from 2.63 ± 0.7 to 1.32 ±0.6 Hz (*P*<0.05, Student’s t test, Fig 5E (Mg^2+^) and Fig 5F) and the amplitude from 12.45 ± 2.6 to 6.51 ± 2.2 pA (*P*<0.05, Fig 5E (Mg^2+^), Fig 5G and 5H) in the RTT CA1 neurons.

Significant changes in the frequency and amplitude in the above results imply presynaptic and postsynaptic effects of pH variations. Additionally, elevated magnesium also reduced the frequency and amplitude of mEPSCs, asserting its effect on synaptic release mechanisms as well as on postsynaptic receptors.

### Back-propagating action potentials and surface potential effects

**Fig 6:**
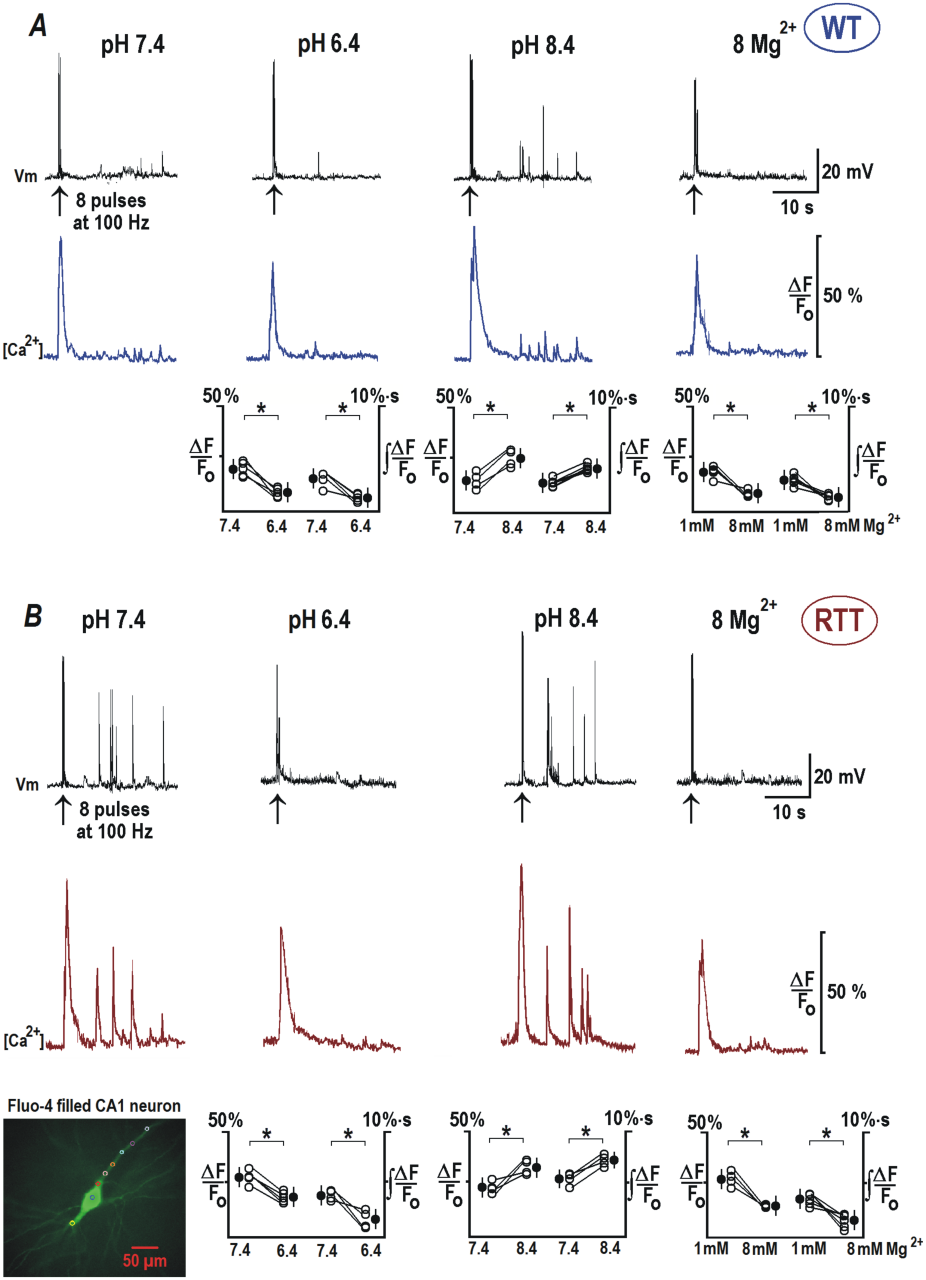
Back-propagating potentials are modulated by extracellular pH and Mg^2+^. Sample recordings show the responses of CA1 cells from RTT (***A***) and WT animals (***B***) as indicated. bAPs were evoked by 8 pulses delivered at 100 Hz. Calcium changes were measured as relative changes in fluorescence of fluo-4 added to pipette solution. Upper traces in each panel show voltage trajectories and the lower traces indicate calcium changes in the apical dendrite (100 μm apart from the cell soma, see the inset, left bottom). In the control ACSF (pH 7.4), stimulation triggered normally several discharges within a minute after bAPs, which were mirrored by the calcium transients (lower violet traces). The responses in RTT CA1 neurons (***B****)* showed marked enhancement. In ACSF with pH 8.4, the post-stimulation activities in WT and RTT were augmented (in RTT to much bigger extent). Lowering external pH to 6.4 and 8 mM Mg^2+^ markedly depressed ‘after-discharges’. Group summaries in the lower part of (***A***) and (***B***) represent peak calcium amplitudes during bAPs and the integral during after-discharges. The data was evaluated before and 2 min after the exchange of bathing solutions. The inset in the left bottom corner shows a typical CA1 neuron filled with fluo-4.

To assess and compare dendritic integration in the CA1 neurons of WT and RTT, we measured back-propagating action potentials (bAPs) in patch-clamp recordings combined with intracellular calcium imaging using fluo-4. The responses were measured electrically at the soma, and as calcium changes in the apical dendrites. Calcium responses were evaluated as relative increases in fluo-4 fluorescence (= ΔF/F_o_ ~ [Ca]) and changes in the integral of ΔF/F_o_, obtained 10 s after trains of 8 pulses at 100 Hz. In the control (pH 7.4), brief stimulation repeatedly produced ‘after-discharges’, that consisted of several APs and were mirrored by calcium transients in the soma and dendrites (Fig 6A and 6B). At pH 8.4, bAP-evoked activities were reinforced in both genotypes. Conversely, the acidic solutions and elevated Mg^2+^ abolished the after-discharges. The responses in RTT and WT neurons showed similar trends but had qualitative differences that can be explained by differential activities of HCN and VSCC channels in these cell types.

In the WT CA1 neurons (Fig 6A) bAP increased a ΔF/F_o_ by 15.02 ± 6.1% at pH 7.4. Area under the curve of ‘Ca^2+^ after-discharges’ had a magnitude of 2.57 ± 0.66%·s. A decrease in the extracellular pH to 6.4 (n = 6) significantly decreased the amplitude of Ca^2+^ transient to 10.06 ± 4.1% (*P*<0.05, Student’s t test) from the control amplitude of 17.39 ± 4.7%. Integral of after-discharges also decreased significantly from the control value to 1.17 ± 0.43%·s (*P*<0.05, Student’s t test). Incubation with ACSF at pH 8.4 (n = 7) resulted in a Ca^2+^ increase in response to bAP of 24.95 ± 5.34% (*P*<0.05, Student’s t test) and a mean integral of Ca^2+^ after-discharges was 3.42 ± 0.7%·s (*P*<0.05, Student’s t test). Exposure to 8 mM Mg^2+^ (n = 5) had effects similar to acidification and reduced the amplitude of bAP induced Ca^2+^ transient to 12.56 ± 4.8% (*P*<0.05, Student’s t test) from a control value of 22.43 ± 5.4%. The integral value of after-discharge induced Ca^2+^ transients diminished from a control value of 2.64 ± 0.42 to 1.21 ± 0.38%·s (*P*<0.05, Student’s t test).

RTT CA1 neurons (Fig 6B) displayed evidently increased sensitivity to bAP induction, characterized by higher Ca^2+^ responses and more intensive after-discharges. Ca^2+^ transient in response to bAPs had a significantly higher amplitude of 25.27 ± 6.3% (*P*<0.05, Mann-Whitney U test), in comparison to WT at normal extracellular pH. Integral of Ca^2+^after-discharges also had an increased magnitude of 4.92 ± 0.62%·s (*P*<0.05, Mann-Whitney U test). Reducing pH to 6.4 (n = 6) diminished the amplitude of the Ca^2+^ transients from a basal value of 25.28 ± 6.6 to 19.83 ± 5.7% (*P*<0.05, Student’s t test). Acidification almost completely abolished the generation of AP after-discharges and related Ca^2+^ responses (from 3.73 ± 0.72 to 1.34 ± 0.7%·s (*P*<0.05, Student’s t test). Switching to pH 8.4 (n = 7) had a further stimulatory effect on the amplitude of bAP induced Ca^2+^ signal (34.76 ± 6.51%, *P*<0.05, Student’s t test) and the integral of Ca^2+^ transients in response to after-discharges (6.84 ± 0.72%·s, *P*<0.05, Student’s t test). 8 mM Mg^2+^ application (n = 7) emulated a similar effect to that of acidification and significantly reduced the amplitude of bAP induced Ca^2+^ transient (from 27.02 ± 5.8 to 13.14 ± 5.3%, *P*<0.05, Student’s t test) and partially abolished the subsequent after-discharges (the integral Ca^2+^ transient decreased to 1.14 ± 0.46 from 2.67± 0.62%·s, *P*<0.05, Student’s t test).

The dendritic signaling was also distinctly perturbed in the RTT CA1 neurons as shown by the above data. Unlike in the WT neurons, the bAPs often caused after-discharges (and Ca^2+^ transients) in the RTT CA1 dendrites. These discharges were exacerbated in alkaline condition. On the contrary acidic pH and elevated magnesium were able to attenuate these spontaneous discharges and calcium increase.

## Discussion

Neurotransmission is extremely sensitive to pH variation, and the regulation of pH is of utmost importance for the proper functioning of the central nervous system. The pH of the body fluids is readily altered during hyper/hypoventilation leading to respiratory alkalosis or acidosis respectively. Hyperventilation provoked alkalosis of extracellular environment is shown to cause carpopedal spasms [41, 42] and seizure [43]. Irregular breathing [44], apnoeas [45, 46] and hyperventilation [47], are typical symptoms of Rett syndrome patients. Such respiration related anomalies and resulting alkalinization of extracellular milieu can contribute to the induction of epilepsy, another prevalent feature of RTT patients [44]; by promoting neuronal hyperexcitability.

Our data elucidate the biophysical mechanisms (variations in subthreshold channel currents) that can underlie enhanced excitability of neurons in RTT and uncover the vulnerability of RTT neuronal activity to alkaline environment. The results are also helpful to devise treatment strategies to abate hyperexcitability and epileptic attacks. For this study, organotypic slices from the hippocampus of neonatal mice after 7 days (P10) in culture (prepared at age P3) were used. Compelling evidence from different laboratories [23, 31 and 48] convincingly demonstrate that this ex vivo preparation closely mimic both intrinsic activity in the hippocampus and its development. Use of organotypic slices enabled the transduction with viral mediated glutamate sensor iGluSnFR to image glutamate changes in situ.

The pivotal hypothesis of this study is that the increased excitability in RTT patients can be exaggerated by extracellular alkalization due to respiratory malfunctions and that the RTT neurons are more vulnerable to such changes. This is evidently shown by our alkalinization experiments, where it increases excitability (Fig 1B) and enhances generation of glutamate spikes (Fig 3B). Acidic extracellular pH and elevated Mg^2+^ salvaged RTT CA1 neurons from dysfunctional burst firing (Figs 1B and 4A) and glutamate accumulation (Figs 3A and 4B). These modifications are concordant with the shifts in the activation curves of hyperpolarization-activated channels and voltage sensitive calcium channels, which designate the corresponding alterations in the cell membrane surface potential.

### Role of HCN and VSCC in RTT hyperexcitability

HCN and some classes of VSCC channels are active at ‘subthreshold’ membrane potentials [19–21]. The modulation of these channels can readily alter neuronal activity and drive the membrane potential towards AP threshold [49, 50]. Surface potential change related effects were qualitatively similar in CA1 cells from the hippocampus of WT and RTT animals, but the ‘basal physiological conditions’ among them were very different. In RTT neurons the HCN currents were smaller, as their activation was hyperpolarization-shifted (Fig 2B and 2D, left). The same hyperpolarization shift in gating was seen for VSCC currents too, which made their amplitude higher in response to same membrane depolarization (Fig 2B and 2D, right). Such a combination of conductance changes can act concomitantly to boost the neuronal firing and can culminate in higher susceptibility of RTT hippocampus to epilepsy. Correspondingly, in the slices from RTT mice, we frequently observed spontaneous glutamate spikes and epileptic-like burst firing, which were virtually absent in WT slices. A concerted action of HCN and VSCC gating shift can explain these changes in excitability of RTT CA1 neurons. The isolation and analysis of individual currents conducted by respective channel subtypes contributing to hyperexcitability requires further detailed experiments.

A parallel shift in the activation curves of HCN and VSCC channels can act synergistically to increase excitability of RTT CA1 neurons. Under such circumstances the VSCC channels start opening at voltages closer to the resting potential and promote excitatory drive. HCN channels are shown to exert dichotomous effects on the neuronal excitability. On one hand, they provide depolarization drive at voltages more negative than its reversal potential (-40 mV, [51]); and on the other hand, HCN activation decreases membrane resistance, and the resulting ‘shunting effect’ decreases membrane excitability. Recent studies indicate that the second effect is dominant in CA1 neurons, and an active HCN-mediated shunt conductance normally dampens neuronal excitability [38–40]. Therefore a decreased contribution of HCN channels in RTT CA1 neurons can improve temporal synchrony at the dendrites and render them more responsive to synaptic inputs [40]. Nearly identical shift in the activation curves of HCN and VSCC currents hint a similar underlying mechanism, which can be the change in the surface potential.

Epileptic seizures are often found to be associated with the loss of expression and function of HCN channels [52]. Their function as ‘voltage-absorber’ suppresses excitability of pyramidal neurons by decreasing the dendritic input resistance [53]. Similarly, calcium channels play an important role as a trigger for the presynaptic glutamate release and postsynaptic integration. A combined modulation of HCN and VSCC channel activities is sufficient to accomplish the increased excitability observed in the RTT neurons and its changes by external pH variations (Fig 1). The shift of voltage-dependent activation to opposite directions by acidic and alkaline ACSF (Fig 2D) affirms the concept of how surface potential modulates the voltage-dependent properties of ionic channels [14]. Even though the shifts were <10 mV in absolute value (Figs 2D and 4D), they are sufficient to substantially alter the amplitudes of HCN and VSCC currents at subthreshold potentials. The absolute changes imposed by alkaline and acidic solutions were nearly equal, and seems to correspond to a pK_H_ of negative surface charges close to the normal extracellular pH (7.4). This is identical to the pK_H_ value reported for HCN channels in rod photoreceptors (pK_H_ = 7.29, [32]) and VSCC in rat thalamic relay neurons (pK_H_ = 6.9, [54]). A shift in *V*_*1/2*_ *≈* 5 mV evoked by 8 mM Mg^2+^ is also in the range of expected changes in the surface potential [15].

Both pre- and postsynaptic functions are extremely sensitive to pH changes. The opening and conductance of presynaptic VSCC critically depend on pH [55]. Intracellular calcium release (through IP_3_ and Ryanodine receptors) at the presynaptic endings, which can augment the neurotransmitter release, is also shown to be pH sensitive [56, 57]. Significant modulation of the biophysical properties of pre-and postsynaptic VSCCs occur during pH changes. The reduction in calcium channel activity during acidification cannot only be attributed to the pore blockade by protons [58]. Acidic pH is also shown to amplify HCN current and shift the activation curve to more positive potential in vomeronasal neurons [59]. Our results indicate that one of the factors contributing to such changes can be the shifts in cell membrane surface potential. Interstitial pH is also crucial to AMPA and NMDA receptor signaling. Protons inhibit AMPA [60] and NMDA [61] receptor currents, whereas alkaline pH elevated their conductance and increased the excitability [62]. As depicted in Fig 3, the decrease in the glutamate signal in response to pH 6.4 can be mediated by the inhibition of conductance of AMPA and NMDA receptor conductance. Protons also enhance the desensitization of AMPA receptors [60] and underlie the decrease in the frequency of EPSCs and the corresponding glutamate spikes. Alkaline pH (8.4), on the other hand, provides relief from proton block and thereby increases the frequency of EPSCs and glutamate spikes.

pH variation is shown not to significantly influence the rate of glutamate uptake by glia as reported by Federovich et al. [63]. Recent evidence [64] shows that glial abnormalities may well be involved in the pathogenesis of RTT but the role of glia in causing abnormal glutamate homeostasis is not yet known. We performed experiments to activate microglia in WT and RTT slices by incubation with lipopolysaccharides, but did not find any changes in the glutamate handling in both genotypes (unpublished data).

Our data support the prospect that HCN channels can serve as major targets to tackle RTT hyperexcitability. These channels are crucial in the control of neuronal discharge patterns and heart rate [65]. HCN channels are reported to regulate excitatory-inhibitory imbalance in Fragile X Syndrome, an autism spectrum disorder, where their lower expression lead to enhanced dendritic excitability [66]. HCN1 channels are also proven to constrain dendritic calcium spikes in CA1 dendrites [38], and are also involved in the hippocampal maturation and network responses [67], displaying developmental plasticity in axonal and presynaptic compartments that modulates synaptic adaptability. A previous theoretical analysis shows that the decrease of dendritic HCN conductance makes EPSPs faster and bigger [39]. This concurs with our findings that HCN channel current is smaller and the EPSC amplitudes are consecutively bigger in RTT CA1 neurons. HCN current amplitude decreases further at pH 8.4; increases substantially at pH 6.4 and elevated Mg^2+^ (Figs 2 and 4). Thus, at pH 8.4, EPSCs accelerated and gained amplitude in response to the decrease in HCN current. At pH 6.4 and elevated Mg^2+^, EPSCs were smaller than that recorded at pH 7.4 (Fig 5, and the insets on the right), in parallel to an increase in HCN current.

HCN channels were previously proposed as a target for anticonvulsant, anesthetic and analgesic drugs [68]. One candidate drug to weaken neuronal excitability is lamotrigine, which upregulates HCN channel activity through an approximately 10 mV depolarization shift. It has already been used as an antiepileptic drug to treat convulsions in Rett patients [69], and shown to markedly decrease stereotyped hand movements and autistic behavior.

### Mg^2+^ is a candidate medicament to constrain hyperexcitability in RTT

One of the hallmarks of Rett syndrome is irregular cycles of hyperventilation with concomitant hypocapnia, often followed by apnoea (breath holding) and unconsciousness [10]. Mg^2+^ is crucially involved in the control of neuronal and muscle excitability and its deficiency leads to muscle and neuronal disorders such as cramps and psychiatric problems [70]. Low magnesium enhances excitability, and surface potential change is proposed to play an essential part in the initiation of seizures in low Mg^2+^ and Ca^2+^ environments [71].

Egger et al. [22] prescribed magnesium orotate or citrate orally to Rett patients (4 mg to 10 mg/kg). This treatment decreased the daily episodes of cyanosis and fewer episodes of hyperventilation occurred in the patients. All Rett patients in the above study had respiratory alkalosis before magnesium was administered. When magnesium was stopped, over-breathing recurred, which decreased after magnesium was reintroduced. Upon magnesium treatment, even the previously exhibited hand stereotypies and agitated behavior subsided. The authors suggest that magnesium diminish the activity of NMDA receptors. However, in our experiments, irreversible blockade of NMDA receptor with 50 μM MK-801 and reversible antagonist AP-5 (50 μM) did not produce significant reduction in the hyperexcitability of RTT CA1 neurons (n = 4, S4A and S4B Fig). The findings in this study presume that the easier induction of hyperexcitability in RTT and the suppressing effect of Mg^2+^ are mediated by changes in the surface potential. 8 mM Mg^2+^ efficiently counters the shift in the gating and as a result, the activation curves of I_HCN_ and I_VSCC_ in RTT CA1 neurons are restored close to normal gating, as in WT. Elevated Mg^2+^ evidently increases HCN conductance, which act as a shunt and decreases calcium conductance at subthreshold potentials. Both effects help to restore the basal excitability and glutamate release in RTT hippocampus and bring these characteristics akin to WT (Fig 4). Acidosis induction in our experiments acted similar to Mg^2+^. But, artificial induction of acidosis (e.g. even a respiratory one invoked by breathing into a paper bag) in patients by increasing HCO_3_^−^ is inadvisable, as it can have adverse cardiovascular and neurological effects. Elevating Ca^2+^ to stabilize surface potential also have drawbacks, as in addition to the surface charge neutralization, it can have aberrant effects on neuronal signaling and muscle contraction. Mg^2+^ treatment in our experiments efficiently attenuated the hyperexcitability and the regenerative glutamate release in RTT slices. It also rectifies the synaptic activity and dendritic conductance in RTT CA1 neurons, as shown by its effects on mEPSCs and back-propagating potentials (Figs 5 and 6 respectively).

## Conclusion

Patch-clamp recordings and glutamate imaging experiments in the CA1 area of hippocampus recapitulate the importance of surface potential in shaping neuronal excitability, and accentuate its specific implication in Rett syndrome hyperexcitability. The results also reaffirm and underscore previous empirical clinical findings. The paradigms can be adopted to treat symptoms such as epilepsy, apnoea and motor dysfunctions in RTT.

## Supporting information

S1 Fig Neuronal and glial glutamate sensors reported identical spatiotemporal signals. Neuronal and glial targeted glutamate sensor signals were very similar in kinetics in the WT slices (***A***, and ***B*)** and RTT slices **(*D*** and ***E***) during control conditions and during pH changes. Both sensors reported analogous signals under normal and 8 mM Mg^2+^ concentrations in both genotypes (***C*** and ***F***).
(TIF)

S2 Fig Comparison of electrophysiological properties of CA1 neurons of WT and Mecp2 knock out. **(*A*)** Input resistances of WT and RTT CA1 neurons were measured in response to current pulses (500 pA, 500 ms). There was no statistically significant difference between the input resistance of WT and RTT CA1 neurons under control conditions (‘n’ represents number of neurons analyzed). RTT CA1 neuron input resistance reduced significantly (*P<0.05, Student’s t test) during external pH change to 6.4 and showed no changes at pH 8.4. Perfusion with 8 mM Mg^2+^ did not change the input resistance of RTT CA1 neurons significantly. (***B***) The whole cell capacitance were measured from the capacitance transients in response to voltage pulses (to ± 10mV) and showed no statistically significant differences between CA1 neurons from WT and RTT. (***C***) The resting membrane potentials were measured during current clamp recordings, and WT and RTT CA1 neurons showed no statistically significant differences in their resting membrane potentials.
(TIF)

S3 Fig Action potential kinetics of WT and RTT CA1 neurons. (***A***) AP rise times in CA1 neurons from WT, RTT CA1 neurons during control conditions and RTT CA1 neurons during exposure to solutions with pH 6.4, 8.4 or with 8 mM Mg^2+^. (***B***) Comparison of AP decay times of CA1 neurons from WT, RTT CA1 neurons during control conditions and RTT CA1 neurons during exposure to solutions with pH 6.4, 8.4 or with 8 mM Mg^2+^. (***C***) Comparison of AP threshold of CA1 neurons from WT, RTT CA1 neurons during control conditions and RTT CA1 neurons during exposure to ACSF with pH 6.4, 8.4 or with 8 mM Mg^2+^.
(TIF)

S4 Fig NMDA receptor blockade did not suppress hyperexcitability in RTT CA1 neurons. (***A***) Application of NMDA receptor blocker D-AP5 (50 μM) to spontaneously active RTT CA1 neurons had no inhibitory effect on the bursting activity. (***B***) Similarly irreversible NMDA receptor antagonist (open channel) MK-801 (10 μM) also did not change the excitability pattern of the RTT CA1 neurons.
(TIF)
