# Supplementary figures and images for: Rescue of hyperexcitability in hippocampal CA1 neurons from Mecp2 (-/y) mouse through surface potential neutralization
Source: PLoS One. 2018 Apr 5;13(4):e0195094. doi: 10.1371/journal.pone.0195094 (PMC5886422; doi:10.1371/journal.pone.0195094)

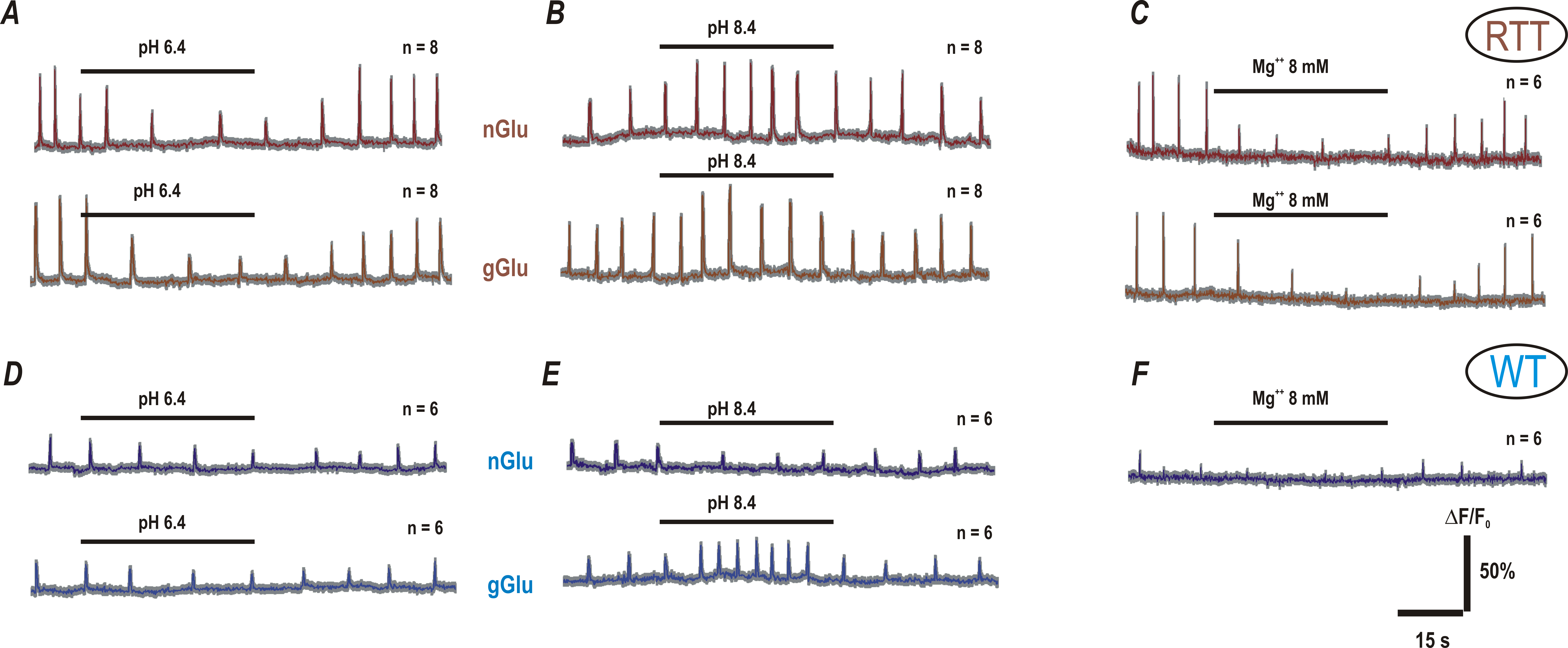

Supplement: S1 Fig — Neuronal and glial targeted glutamate sensor signals were very similar in kinetics in the WT slices (A, and B) and RTT slices (D and E) during control conditions and during pH changes. Both sensors reported analogous signals under normal and 8 mM Mg2+ concentrations in both genotypes (C and F). (TIF) [file pone.0195094.s001.tif]

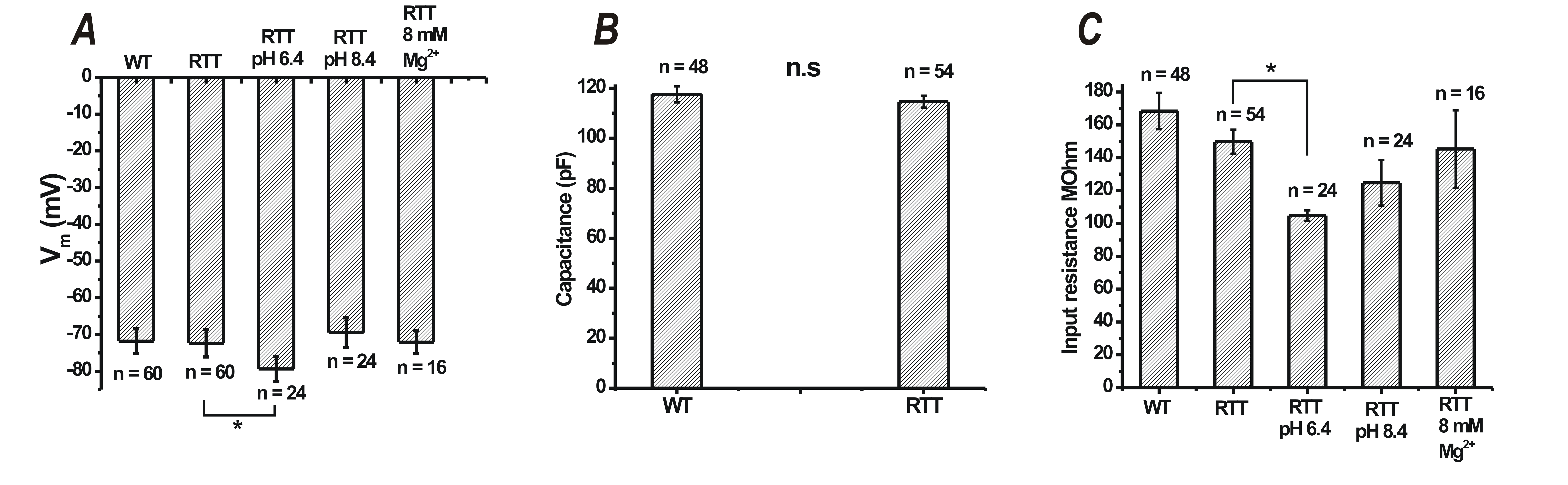

Supplement: S2 Fig — (A) Input resistances of WT and RTT CA1 neurons were measured in response to current pulses (500 pA, 500 ms). There was no statistically significant difference between the input resistance of WT and RTT CA1 neurons under control conditions (‘n’ represents number of neurons analyzed). RTT CA1 neuron input resistance reduced significantly (*P<0.05, Student’s t test) during external pH change to 6.4 and showed no changes at pH 8.4. Perfusion with 8 mM Mg2+ did not change the input resistance of RTT CA1 neurons significantly. (B) The whole cell capacitance were measured from the capacitance transients in response to voltage pulses (to ± 10mV) and showed no statistically significant differences between CA1 neurons from WT and RTT. (C) The resting membrane potentials were measured during current clamp recordings, and WT and RTT CA1 neurons showed no statistically significant differences in their resting membrane potentials. (TIF) [file pone.0195094.s002.tif]

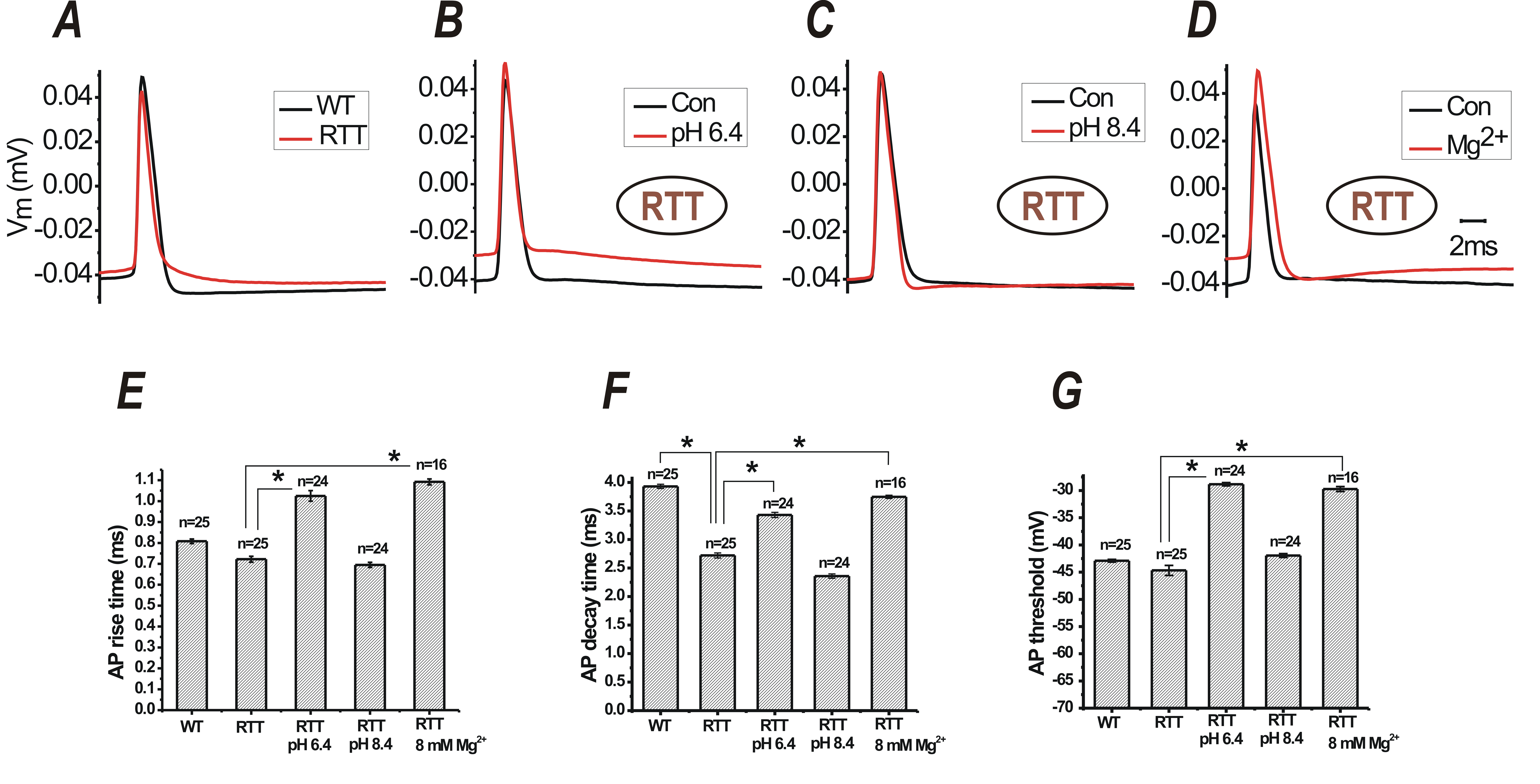

Supplement: S3 Fig — (A) AP rise times in CA1 neurons from WT, RTT CA1 neurons during control conditions and RTT CA1 neurons during exposure to solutions with pH 6.4, 8.4 or with 8 mM Mg2+. (B) Comparison of AP decay times of CA1 neurons from WT, RTT CA1 neurons during control conditions and RTT CA1 neurons during exposure to solutions with pH 6.4, 8.4 or with 8 mM Mg2+. (C) Comparison of AP threshold of CA1 neurons from WT, RTT CA1 neurons during control conditions and RTT CA1 neurons during exposure to ACSF with pH 6.4, 8.4 or with 8 mM Mg2+. (TIF) [file pone.0195094.s003.tif]

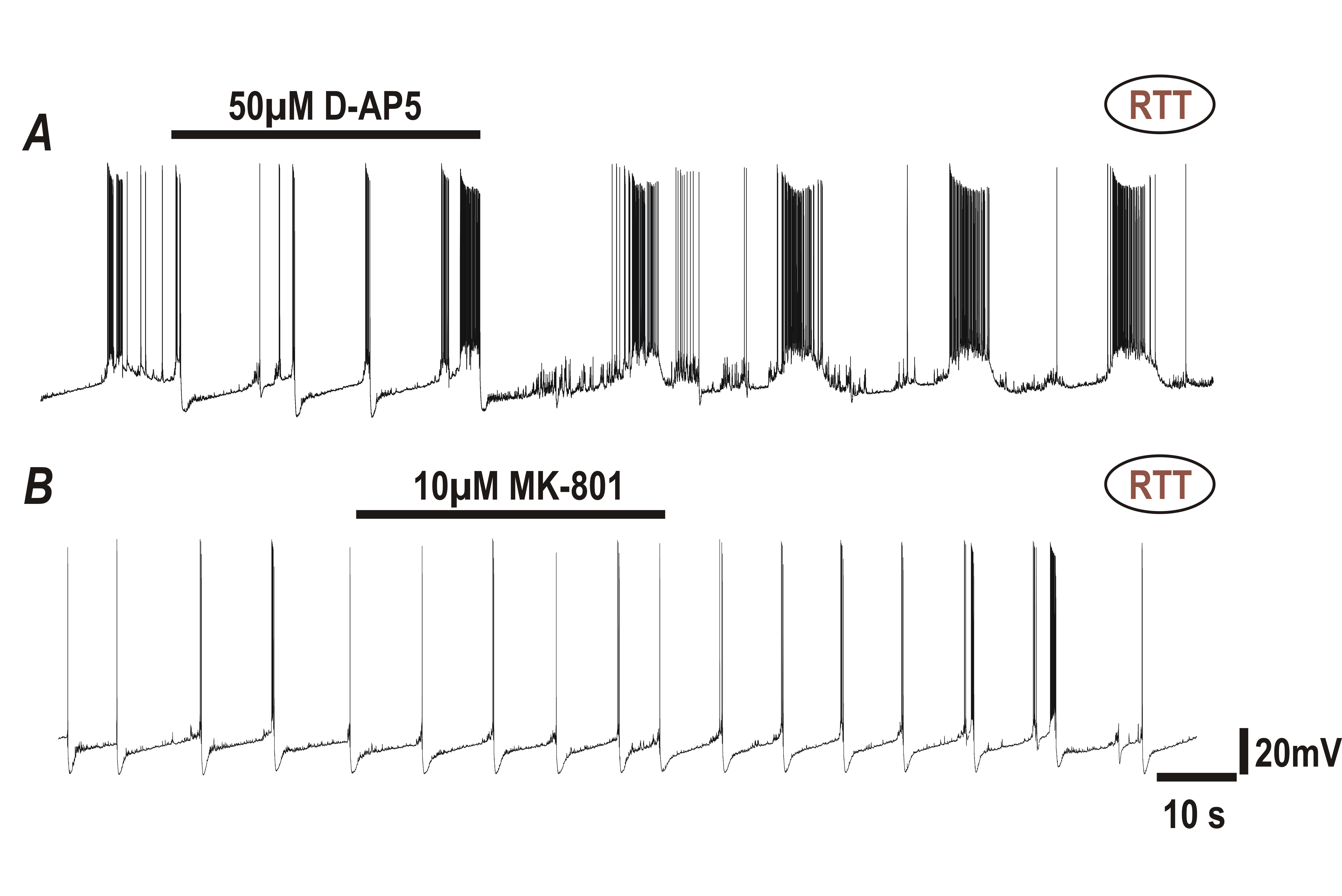

Supplement: S4 Fig — (A) Application of NMDA receptor blocker D-AP5 (50 μM) to spontaneously active RTT CA1 neurons had no inhibitory effect on the bursting activity. (B) Similarly irreversible NMDA receptor antagonist (open channel) MK-801 (10 μM) also did not change the excitability pattern of the RTT CA1 neurons. (TIF) [file pone.0195094.s004.tif]
